# Clinical characteristics of glaucoma patients with various risk factors

**DOI:** 10.1186/s12886-022-02587-5

**Published:** 2022-09-19

**Authors:** Kazuko Omodaka, Tsutomu Kikawa, Sayaka Kabakura, Noriko Himori, Satoru Tsuda, Takahiro Ninomiya, Naoki Takahashi, Kyongsun Pak, Noriyasu Takeda, Masahiro Akiba, Toru Nakazawa

**Affiliations:** 1 Department of Ophthalmology, Tohoku University, Graduate School of Medicine, Sendai, Japan; 2 Department of Ophthalmic Imaging and Information Analytics, Tohoku University, Graduate School of Medicine, Sendai, Japan; 3 R and D Division, Topcon Corporation, Tokyo, Japan; 4 Department of Aging Vision Healthcare, Tohoku University Graduate School of Biomedical Engineering, Sendai, Japan; 5 Division of Biostatistics, Center for Clinical Research, National Center for Child Health and Development, Tokyo, Japan; 6 Cloud-Based Eye Disease Diagnosis Joint Research Team, Riken, Wako, Japan

**Keywords:** Risk factors, Optical coherence tomography, Individual treatment for glaucoma, Blood pressure, Glaucoma

## Abstract

**Background:**

Glaucoma is multifactorial, but the interrelationship between risk factors and structural changes remains unclear. Here, we adjusted for confounding factors in glaucoma patients with differing risk factors, and compared differences in structure and susceptible areas in the optic disc and macula.

**Methods:**

In 458 eyes with glaucoma, we determined confounding factors for intraocular pressure (IOP), central corneal thickness (CCT), axial length (AL), LSFG-measured ocular blood flow (OBF), which was assessed with laser speckle flowgraphy-measured mean blur rate in the tissue area (MT) of the optic nerve head, biological antioxidant potential (BAP), and systemic abnormalities in diastolic blood pressure (dBP). To compensate for measurement bias, we also analyzed corrected IOP (cIOP; corrected for CCT) and corrected MT (cMT; corrected for age, weighted retinal ganglion cell count, and AL). Then, we determined the distribution of these parameters in low-, middle-, and high-value subgroups and compared them with the Kruskal–Wallis test. Pairwise comparisons used the Steel–Dwass test.

**Results:**

The high-cIOP subgroup had significantly worse mean deviation (MD), temporal, superior, and inferior loss of circumpapillary retinal nerve fiber layer thickness (cpRNFLT), and large cupping. The low-CCT subgroup had temporal cpRNFLT loss; the high-CCT subgroup had low cup volume. The high-AL subgroup had macular ganglion cell complex thickness (GCCT) loss; the low-AL subgroup had temporal cpRNFLT loss. The high-systemic-dBP subgroup had worse MD, total, superior, and inferior cpRNFLT loss and macular GCCT loss. The low-BAP subgroup had more male patients, higher dBP, and cpRNFLT loss in the 10 o’clock area. The high-OBF subgroup had higher total, superior and temporal cpRNFLT and macular GCCT.

**Conclusions:**

Structural changes and local susceptibility to glaucomatous damage show unique variations in patients with different risk factors, which might suggest that specific risk factors induce specific types of pathogenesis and corresponding glaucoma phenotypes. Our study may open new avenues for the development of precision medicine for glaucoma.

## Background

Glaucoma is a multifactorial disease [1] with various reported risk factors, including high intraocular pressure (IOP), aging, myopia, [2] family history, [3] thin corneal central thickness (CCT) and corneal hysteresis, low ocular blood flow (OBF), abnormality of the lamina cribrosa, oxidative stress, neuroinflammation, and lifestyle factors, such as diabetes, sleep apnea, diet, and smoking [1, 4–9]. Lowering IOP is the only evidence-based treatment for preventing glaucoma progression, [10] but other risk factors are likely also involved in retinal ganglion cell (RGC) loss in glaucoma, acting via various pathways. IOP-lowering treatments are highly developed, including innovative eye drops with novel IOP-lowering mechanisms, minimally invasive surgical devices, and laser treatment. Nevertheless, the increasing number of patients with glaucoma-induced blindness is a serious problem in aging societies [11]. Therefore, there is a need for new treatments based on the pathophysiology of glaucoma. Specifically, there is an unmet medical need to prevent blindness in patients with glaucoma caused by non-IOP factors.

Previous epidemiological studies have shown that systemic OBF is deeply involved in the prevalence, development, and progression of glaucoma [1, 5, 12, 13]. Laser speckle flowgraphy (LSFG) clearly shows that optic nerve head (ONH) tissue-area mean blur rate (MT) significantly decreases in preperimetric glaucoma (PPG), [14] and that the peak of the LSFG waveform is delayed in early glaucoma [15]. We have also found, in an observational study of PPG, that low OBF is an important risk factor for glaucoma progression [16]. The loss of retinal nerve fibers reduces capillary density in the retinal nerve fiber layer (RNFL), a phenomenon that has led to much discussion as to whether decreased OBF is the primary or secondary event in glaucoma, i.e., whether it occurs before or after the onset of visual field defects. Recently, we made two very interesting findings. First, in glaucoma patients that we identified as having primary decreased OBF by adjusting for confounding factors, low OBF was associated with a larger optic disc cupping and a greater incidence of damage in the macula [17]. Second, we found that older patients with a higher pulse rate were at greater risk of a primary reduction in OBF, and that this reduction preceded a decrease in circumpapillary RNFL thickness (cpRNFLT) in the superior and temporal quadrants [18]. These findings led us to theorize that different risk factors are associated with different pathophysiologies and disc phenotypes, as well as different vulnerabilities to glaucomatous lesions.

In the present study, we investigated how various measurable risk factors for glaucoma, including pre-treatment IOP (preIOP), CCT, oxidative stress, axial length (AL), systemic dBP, and MT, induced differing patterns of glaucomatous structural damage. These risk factors were, in part, correlated to each other, so we divided the subjects into three groups based on high, middle, and low values of corrected parameters for each of the various risk factors (i.e., the total number of patients was divided into high, middle, and low preIOP groups, high, middle, and low CCT groups, etc.) and performed a multiple regression analysis to isolate the exact role of each risk factor. Then, we compared structural differences in glaucomatous damage in the tripartite groups. These findings may help uncover new methods for the clinical management of glaucoma based on specific risk factors and allow the development of new treatments based on IOP-independent risk factors to address unmet medical needs.

## Materials and methods

This study enrolled 458 eyes of 458 patients with open-angle glaucoma (OAG) (male/female: 244/214, age: 56.7 ± 14.4 years old, preIOP: 18.8 ± 7.0 mmHg, mean deviation: -8.9 ± 7.8 dB, cpRNFLT: 71.4 ± 17.5 μm; Table 1). The inclusion criteria were: (1) a diagnosis of OAG, including either primary open-angle glaucoma (POAG) or normal-tension glaucoma (NTG) and (2) a glaucomatous visual field meeting the Anderson-Patella classification; i.e., with one or more of the following: (1) a cluster of three points with probabilities of < 5% on the pattern deviation map in at least one hemifield (including ≥ 1 point with probability of < 1% or a cluster of two points with a probability of < 1%, (2) glaucomatous hemifield test results outside the normal limits or (3) a pattern standard deviation beyond 95% of normal limits, as confirmed in at least 2 reliable examinations. Preperimetric glaucoma patients were not included in this study. The exclusion criteria were: (1) age younger than 20 years, (2) best-corrected visual acuity (BCVA) less than 0.3, (3) high myopia (i.e., spherical equivalent refraction error <-7.0 diopters), and (4) the presence of non-OAG ocular disease or any other systemic disease affecting the visual field.

The baseline clinical parameters recorded for each patient were age, gender, and refractive error. IOP was measured with Goldman applanation tonometry at the time of the initial diagnosis of OAG, before the use of any medications for glaucoma (preIOP) or post-treatment IOP (postIOP). CCT was measured with anterior-segment OCT (Casia, Tomey Corporation, Nagoya, Japan). Mean deviation (MD) and pattern standard deviation (PSD) were measured with the Humphrey visual field analyzer (HFA) using the Swedish interactive threshold algorithm (SITA)-standard strategy of the 24–2 program. Only reliably measured data were used (i.e., with a fixation loss < 20%, false-positive errors < 15%, and false-negative errors < 33%). The LSFG-NAVI device (Softcare Co., Ltd., Fukutsu, Japan) was used to assess OBF in the ONH by measuring MT, a measurement parameter expressed in arbitrary units. MT does not represent global ocular hemodynamics, as it is only a single parameter captured over a few seconds. However, it has excellent reproducibility [19] and has been shown to be significantly correlated to microsphere-measured OBF in the posterior ciliary artery in primates [20], hydrogen gas clearance–measured OBF, [21] and the severity of glaucomatous damage [22]. In this study, we used MT as a parameter to represent OBF. LSFG images were captured after subjects rested for 10 min in a dark room. Stable systolic and diastolic blood pressure were measured simultaneously with LSFG.

Swept-source OCT (DRI OCT Triton, Topcon Corp., Tokyo, Japan) was used for the assessment of structure. Horizontal disc scans and macular maps (6 × 6 mm, 512 A scans × 256 frames) were obtained and software was used (FastMap Ver. 10.16, Topcon) to identify the boundaries of the retinal nerve fiber layer (RNFL), ganglion cell layer and inner plexiform layer (GCIPL), and ganglion cell complex (GCC) layer. Total, quadrant, and clock-hours cpRNFL thickness and total and hemifield macular GCC thickness were used for the analysis.

We used multi-regression analysis to calculate a corrected MT (cMT) index by normalizing for three confounding factors: age, AL, and a weighted count of retinal ganglion cells (wRGC), as previously described [17]. PreIOP was corrected based on CCT (cIOP).

Blood samples were collected at least 3 h after the last meal and were immediately examined for oxidative stress with a free radical analyzer system (Free Carpe Diem, Wismerll Company Ltd., Tokyo, Japan), according to the manufacturer’s instructions, as previously described [23]. The level of diacron-reactive oxygen metabolites was expressed in U. Carr. and biological antioxidant potential (BAP) was expressed in µmol/L.

This study adhered to the tenets of the Declaration of Helsinki, and the protocols were approved by the Clinical Research Ethics Committee of the Tohoku University Graduate School of Medicine (study 2021-1-430).

### Statistical analysis

This study used a comparison analysis, rather than a linear correlation analysis, for two reasons. First, some risk factors were correlated with each other. To compensate, cMT was corrected for age, AL, and wRGC, and cIOP was corrected for CCT in the multi-regression analysis. Second, some of the measurements we examined have already been reported to be risk factors for glaucoma at both low and high levels. Therefore, we divided the subjects into three equal groups based on their differing risk factors to assess differences in structural changes and local susceptibility to glaucomatous damage in the groups. This analysis used the Kruskal–Wallis test. If there was a significant difference among the subgroups, we performed pairwise comparisons with the Steel–Dwass test. The significance level was set at 5%. Since this study aimed to evaluate the association between each factor in an exploratory manner, multiplicity between groups was adjusted in the Steel–Dwass test, but multiplicity between tests was not considered. All statistical analyses were performed with JMP software (Pro version 16.1.0, SAS Institute Japan Inc., Tokyo, Japan) and R software (version 4.1.1, R Foundation, Vienna, Austria).

## Results

We found a significant association between preIOP and CCT (r = 0.14, p = 0.005), which prompted us to calculate corrected preIOP (cIOP), which corrected for CCT, with a regression analysis. We also calculated corrected MT (cMT), which corrected for age, AL, CCT, and wRGC. We then divided the total group of patients into subgroups for low, middle, and high values of cIOP, CCT, AL, dBP, BAP, and cMT, and compared these three-part subgroups.

The cIOP subgroups differed significantly in dBP, postIOP, MD and PSD (Table 1). We found that dBP in the high cIOP group was significantly higher than the low and middle cIOP groups; PSD was significantly lower and MD was better in the low cIOP group than in the middle and high cIOP groups. The three subgroups had significantly different total, temporal, superior, and inferior cpRNFLT and cpRNFLT in the 1, 5, 6, 7, 8, 9, 10, 11, and 12 o’clock sectors. The high cIOP group had significantly lower temporal, superior, and inferior cpRNFLT and cpRNFLT in the 1, 5, 6, 8, 9, and 10 o’clock sectors. There were no differences in disc area or disc diameter, but in the high cIOP group, rim volume was smaller and the C/D area ratio was larger than in the low and middle cIOP groups. Total, superior, and inferior macular GCC thickness was significantly lower in the high cIOP group.

**Table 1:** Characteristics of glaucoma patients divided into three groups based on low, medium, or high corrected pre-treatment intraocular pressure

Variable			Total (reference) (*n* = 458)	Low cIOP (*n* = 153)	Mid cIOP (*n* = 152)	High cIOP (*n* = 153)	*P* value	*p* value of pairwise comparison test		
								Low—Mid	Low—High	Mid—High
cIOP (mmHg)			18.8 ± 7.0	13.4 ± 1.8	17.5 ± 1.3	25.6 ± 7.9				
Male / female			244 / 214	78 / 75	84 / 68	82 / 71	0.752			
CCT (μm)			516.6 ± 35.7	514.3 ± 34.3	518.2 ± 33.0	517.2 ± 39.4	0.528			
BAP (µmol/L)			2127.3 ± 284.9	2147.28 ± 236.0	2105.8 ± 353.9	2128.5 ± 253.0	0.902			
AL (mm)			25.4 ± 2.1	25.7 ± 1.7	25.4 ± 1.7	25.4 ± 2.0	0.123			
dBP (mmHg)			76.8 ± 13.6	74.0 ± 12.1	76.5 ± 14.8	79.8 ± 13.4	< 0.001 ***	0.414	< 0.001 ***	0.030 *
cMT (arbitrary units)			9.9 ± 2.6	10.0 ± 2.7	10.1 ± 2.8	9.6 ± 2.4	0.149			
postIOP (mmHg)			14.8 ± 4.7	13.0 ± 3.5	14.4 ± 3.2	17.1 ± 5.9	< 0.001 ***	< 0.001 ***	< 0.001 ***	< 0.001 ***
Age (years)			56.7 ± 14.4	55.4 ± 15.4	56.2 ± 14.3	58.5 ± 13.3	0.106			
MD (dB)			-8.9 ± 7.8	-6.7 ± 6.9	-9.0 ± 7.4	-11.1 ± 8.3	< 0.001 ***	0.010 *	< 0.001 ***	0.082
PSD (dB)			8.0 ± 4.4	7.0 ± 4.4	8.5 ± 4.5	8.6 ± 4.1	0.004 **	0.025 *	0.004 **	0.984
Total cpRNFLT (μm)			71.4 ± 17.5	75.8 ± 16.1	72.9 ± 16.7	65.8 ± 18.2	< 0.001 ***	0.436	< 0.001 ***	< 0.001 ***
Quadrant cpRNFLT (μm)	4_Temporal		64.5 ± 20.5	69.1 ± 20.2	67.5 ± 20.3	56.9 ± 19.1	< 0.001 ***	0.727	< 0.001 ***	< 0.001 ***
	4_Superior		85.6 ± 27.3	91.8 ± 25.7	86.5 ± 27.2	78.7 ± 27.5	< 0.001 ***	0.240	< 0.001 ***	0.017 *
	4_Nasal		58.0 ± 16.5	58.7 ± 15.5	58.3 ± 16.7	56.9 ± 17.4	0.704			
	4_Inferior		77.6 ± 28.5	83.2 ± 28.1	79.1 ± 27.3	70.6 ± 28.7	< 0.001 ***	0.349	< 0.001 ***	0.011 *
12-sector clockwise cpRNFLT (μm)	Temporal	8 o’clock	60.9 ± 24.7	64.9 ± 23.1	63.3 ± 22.7	54.5 ± 26.7	< 0.001 ***	0.795	< 0.001 ***	< 0.001 ***
		9 o’clock	61.1 ± 19.2	64.2 ± 17.7	63.8 ± 18.6	55.4 ± 19.9	< 0.001 ***	0.928	< 0.001 ***	< 0.001 ***
		10 o’clock	71.5 ± 28.6	78.2 ± 28.8	75.6 ± 28.0	60.9 ± 25.9	< 0.001 ***	0.587	< 0.001 ***	< 0.001 ***
	Superior	11 o’clock	87.9 ± 39.4	99.4 ± 40.7	87.4 ± 40.1	77.0 ± 34.1	< 0.001 ***	0.032 *	< 0.001 ***	0.070
		12 o’clock	85.8 ± 31.5	90.3 ± 29.2	85.4 ± 31.2	81.7 ± 33.6	0.016 *	0.291	0.012 *	0.354
		1 o’clock	83.1 ± 27.3	85.5 ± 25.8	86.5 ± 28.7	77.4 ± 26.6	0.002 **	0.869	0.015 *	0.003 **
	Nasal	2 o’clock	65.4 ± 22.0	65.6 ± 21.1	66.1 ± 22.4	64.6 ± 22.6	0.928			
		3 o’clock	53.0 ± 17.5	53.9 ± 15.0	53.0 ± 19.7	51.9 ± 17.4	0.342			
		4 o’clock	55.6 ± 17.5	56.7 ± 16.8	55.9 ± 17.3	54.3 ± 18.5	0.422			
	Inferior	5 o’clock	76.4 ± 24.6	79.5 ± 23.8	77.7 ± 23.0	71.9 ± 26.3	0.006 **	0.640	0.008 **	0.043 *
		6 o’clock	83.3 ± 35.8	89.5 ± 34.7	86.2 ± 36.9	74.3 ± 34.1	< 0.001 ***	0.642	< 0.001 ***	0.009 **
		7 o’clock	73.1 ± 39.7	80.4 ± 40.3	73.4 ± 39.0	65.6 ± 38.4	0.004 **	0.209	0.002 **	0.203
Disc topography	Disc area		2.06 ± 0.75	2.05 ± 0.65	2.03 ± 0.82	2.09 ± 0.79	0.385			
	Cup area		1.52 ± 0.78	1.47 ± 0.74	1.47 ± 0.79	1.62 ± 0.81	0.033 *	0.973	0.070	0.057
	Rim area		0.54 ± 0.42	0.58 ± 0.35	0.55 ± 0.4	0.47 ± 0.49	< 0.001 ***	0.584	< 0.001 ***	0.003 **
	Cup volume		0.43 ± 0.36	0.39 ± 0.3	0.42 ± 0.34	0.50 ± 0.42	0.011 *	0.724	0.012 *	0.073
	Rim volume		0.05 ± 0.07	0.06 ± 0.05	0.05 ± 0.06	0.05 ± 0.09	< 0.001 ***	0.566	< 0.001 ***	0.003 **
	C/D area ratio		0.72 ± 0.20	0.69 ± 0.19	0.70 ± 0.19	0.76 ± 0.21	< 0.001 ***	0.750	< 0.001 ***	0.004 **
	Linear C/D ratio		0.84 ± 0.14	0.82 ± 0.14	0.83 ± 0.13	0.86 ± 0.15	< 0.001 ***	0.781	< 0.001 ***	0.004 **
	Vertical C/D ratio		0.86 ± 0.15	0.84 ± 0.15	0.86 ± 0.14	0.89 ± 0.15	< 0.001 ***	0.386	< 0.001 ***	0.020 *
	Disc diameter (V)		1.66 ± 0.25	1.67 ± 0.24	1.66 ± 0.26	1.65 ± 0.26	0.568			
	Disc diameter (H)		1.57 ± 0.31	1.56 ± 0.29	1.54 ± 0.31	1.59 ± 0.32	0.331			
Macular GCC thickness (μm)	Total		78.0 ± 14.7	82.8 ± 13.6	81.5 ± 15.0	75.7 ± 14.6	< 0.001 ***	0.886	< 0.001 ***	< 0.001 ***
	Superior		83.5 ± 17.0	86.4 ± 15.9	85.8 ± 17.3	78.4 ± 16.7	< 0.001 ***	0.915	< 0.001 ***	< 0.001 ***
	Inferior		76.5 ± 15.6	79.2 ± 15.0	77.3 ± 16.4	72.9 ± 14.8	< 0.001 ***	0.549	< 0.001 ***	0.020 *

Asterisks: “*” indicates *p* < 0.05, “**” *P* < 0.01, and “***” *p* < 0.001. *Mid* Middle, *cIOP* Corrected pre-treatment intraocular pressure, *CCT* Central corneal thickness, *BAP* Biological antioxidant potential, *AL* Axial length, *dBP* Dilated blood pressure, *cMT* Corrected mean blur rate in the tissue area of the optic nerve, *postIOP* Post-treatment IOP with applanation tonography, *MD* Mean deviation, measured with the Humphrey visual field analyzer, *PSD* Pattern standard deviation, *C/D* Cup-disc ratio

The CCT subgroups had significant differences in cIOP, postIOP and age, but similar MD (Table 2). The three subgroups had significantly different total and temporal cpRNFLT and cpRNFLT in the 8, 9, and 10 o’clock sectors. The high CCT group had significantly higher temporal cpRNFLT and cpRNFLT in the 8 and 10 o’clock sectors. There were also differences in cup volume in the three groups. Total and superior macular GCC thickness was significantly lower in the low CCT group than in the high CCT group.

**Table 2:** Characteristics of glaucoma patients divided into three groups based on low, medium, or high CCT

Variable			Total (reference) (*n* = 458)	Low CCT (*n* = 153)	Mid CCT (*n* = 152)	High CCT (*n* = 153)	*P* value	*p* value of pairwise comparison test		
								Low—Mid	Low—High	Mid—High
CCT (μm)			516.6 ± 35.7	479.8 ± 18.3	515.1 ± 8.2	554.5 ± 24.5				
Male / female			244 / 214	76 / 77	76 / 76	92 / 61	0.114			
cIOP (mmHg)			18.8 ± 7.0	17.7 ± 5.9	19.4 ± 8.2	19.5 ± 6.6	0.006 **	0.144	0.004 **	0.373
BAP (µmol/L)			2127.3 ± 284.9	2085.6 ± 304.7	2144.3 ± 273.3	2152.1 ± 273.6	0.071			
AL (mm)			25.4 ± 2.1	25.3 ± 1.9	25.3 ± 1.7	25.8 ± 1.9	0.072			
dBP (mmHg)			76.8 ± 13.6	77.3 ± 14.5	76.4 ± 13.5	76.5 ± 13.0	0.941			
cMT (arbitrary units)			9.9 ± 2.6	9.6 ± 2.5	10.2 ± 2.8	10.1 ± 2.6	0.063			
postIOP (mmHg)			14.8 ± 4.7	13.6 ± 3.9	15.1 ± 5.1	15.9 ± 4.7	< 0.001 ***	0.005 **	< 0.001 ***	0.040 *
Age (years)			56.7 ± 14.4	59.2 ± 13.5	57.2 ± 13.9	53.7 ± 15.3	0.008 **	0.542	0.007 **	0.099
MD (dB)			-8.9 ± 7.8	-10.0 ± 8.2	-8.4 ± 7.4	-8.4 ± 7.6	0.179			
PSD (dB)			8.0 ± 4.4	8.2 ± 4.3	8.1 ± 4.6	7.8 ± 4.3	0.672			
Total cpRNFLT (μm)			71.4 ± 17.5	68.9 ± 17.7	71.7 ± 17.9	73.8 ± 16.6	0.021 *	0.321	0.014 *	0.415
Quadrant cpRNFLT (μm)	4_Temporal		64.5 ± 20.5	60.4 ± 18.1	63.4 ± 21.4	69.7 ± 21.0	< 0.001 ***	0.506	< 0.001 ***	0.011 *
	4_Superior		85.6 ± 27.3	82.1 ± 27.9	87.4 ± 28.1	87.4 ± 25.7	0.166			
	4_Nasal		58.0 ± 16.5	57.2 ± 17.2	58.5 ± 16.0	58.3 ± 16.4	0.801			
	4_Inferior		77.6 ± 28.5	75.7 ± 28.7	77.4 ± 28.7	79.7 ± 28.1	0.334			
12-sector clockwisecpRNFLT (μm)	Temporal	8 o’clock	60.9 ± 24.7	57.6 ± 19.9	59.3 ± 29.3	65.6 ± 23.4	0.002 **	0.941	0.004 **	0.016 *
		9 o’clock	61.1 ± 19.2	57.3 ± 15.4	61.5 ± 22.7	64.6 ± 18.1	0.002 **	0.319	< 0.001 ***	0.125
		10 o’clock	71.5 ± 28.6	66.1 ± 26.8	69.6 ± 28.3	78.9 ± 29.2	< 0.001 ***	0.601	< 0.001 ***	0.005 **
	Superior	11 o’clock	87.9 ± 39.4	82.9 ± 38.2	87.7 ± 39.8	93.0 ± 39.6	0.080			
		12 o’clock	85.8 ± 31.5	82.2 ± 31.8	88.5 ± 32.2	86.7 ± 30.4	0.216			
		1 o’clock	83.1 ± 27.3	81.1 ± 28.6	85.8 ± 27.8	82.5 ± 25.2	0.377			
	Nasal	2 o’clock	65.4 ± 22.0	64.3 ± 23.8	66.7 ± 21.9	65.4 ± 20.3	0.718			
		3 o’clock	53.0 ± 17.5	52.1 ± 16.4	52.3 ± 14.5	54.4 ± 20.8	0.441			
		4 o’clock	55.6 ± 17.5	55.3 ± 17.5	56.6 ± 17.7	55.0 ± 17.5	0.828			
	Inferior	5 o’clock	76.4 ± 24.6	75.4 ± 26.7	77.8 ± 23.8	76.0 ± 23.2	0.713			
		6 o’clock	83.3 ± 35.8	80.8 ± 36.3	83.7 ± 35.5	85.4 ± 35.6	0.460			
		7 o’clock	73.1 ± 39.7	70.8 ± 37.9	70.8 ± 39.8	77.7 ± 41.0	0.222			
Disc topography	Disc area		2.06 ± 0.75	2.07 ± 0.87	2.13 ± 0.80	1.97 ± 0.56	0.423			
	Cup area		1.52 ± 0.78	1.53 ± 0.83	1.62 ± 0.85	1.41 ± 0.65	0.154			
	Rim area		0.54 ± 0.42	0.54 ± 0.48	0.51 ± 0.44	0.55 ± 0.33	0.100			
	Cup volume		0.43 ± 0.36	0.43 ± 0.35	0.50 ± 0.44	0.37 ± 0.27	0.018 *	0.174	0.587	0.013 *
	Rim volume		0.05 ± 0.07	0.05 ± 0.06	0.05 ± 0.09	0.05 ± 0.05	0.087			
	C/D area ratio		0.72 ± 0.20	0.72 ± 0.21	0.74 ± 0.20	0.69 ± 0.20	0.059			
	Linear C/D ratio		0.84 ± 0.14	0.84 ± 0.14	0.85 ± 0.14	0.82 ± 0.13	0.062			
	Vertical C/D ratio		0.86 ± 0.15	0.87 ± 0.14	0.87 ± 0.15	0.85 ± 0.15	0.457			
	Disc diameter (V)		1.66 ± 0.25	1.66 ± 0.28	1.68 ± 0.26	1.63 ± 0.22	0.440			
	Disc diameter (H)		1.57 ± 0.31	1.57 ± 0.35	1.60 ± 0.31	1.53 ± 0.25	0.393			
Macular GCC thickness (μm)	Total		78.0 ± 14.7	78.3 ± 13.6	79.4 ± 13.9	82.2 ± 16.3	0.032 *	0.646	0.028 *	0.202
	Superior		83.5 ± 17.0	81.3 ± 15.8	83.0 ± 16.6	86.1 ± 18.3	0.027 *	0.632	0.022 *	0.198
	Inferior		76.5 ± 15.6	75.3 ± 14.7	75.7 ± 15.0	78.4 ± 16.9	0.157			

Asterisks: “*” indicates *p* < 0.05, “**” *P* < 0.01, and “***” *p* < 0.001. *Mid* Middle, *cIOP* Corrected pre-treatment intraocular pressure, *CCT* Central corneal thickness, *BAP* Biological antioxidant potential, *AL* Axial length, *dBP* Dilated blood pressure, *cMT* Corrected mean blur rate in the tissue area of the optic nerve, *postIOP* Post-treatment IOP with applanation tonography, *MD* Mean deviation, measured with the Humphrey visual field analyzer, *PSD* Pattern standard deviation, *C/D* Cup-disc ratio

The AL subgroups had significant differences in sex, dBP and age (Table 3). We found that dBP in the low AL group was significantly higher than in the middle and high AL groups. Age was significantly higher in the low AL group than in the middle and high AL groups. The three groups had significantly different temporal cpRNFLT and cpRNFLT in the 8, 9, and 10 o’clock sectors. The low AL group had significantly lower temporal cpRNFLT and cpRNFLT in the 8, 9, and 10 o’clock sectors. There were no differences in disc area, but in the low AL group, rim volume was smaller. Total and inferior macular GCC thickness was significantly lower in the high AL group than in the middle AL group.

**Table 3:** Characteristics of glaucoma patients divided into three groups based on low, medium, or high AL

Variable			Total (reference) (*n* = 458)	Low AL (*n* = 153)	Mid AL (*n* = 152)	High AL (*n* = 153)	*P* value	*p* value of pairwise comparison test		
								Low—Mid	Low—High	Mid—High
AL (mm)			25.4 ± 2.1	23.5 ± 0.7	25.4 ± 0.6	27.5 ± 1.1				
Male / female			244 / 214	66 / 87	83 / 69	95 / 58	0.004 **			
cIOP (mmHg)			18.8 ± 7.0	19.4 ± 7.1	18.9 ± 7.4	18.2 ± 6.4	0.340			
CCT (μm)			516.6 ± 35.7	514.0 ± 38.0	517.4 ± 34.6	518.3 ± 34.4	0.158			
BAP (µmol/L)			2127.3 ± 284.9	2131.2 ± 327.4	2148.5 ± 210.5	2102.6 ± 303.3	0.548			
dBP (mmHg)			76.8 ± 13.6	76.1 ± 15.1	74.8 ± 12.7	79.4 ± 12.7	0.007 **	0.824	0.046 *	0.008 **
cMT (arbitrary units)			9.9 ± 2.6	10.0 ± 2.5	9.9 ± 2.6	9.9 ± 2.8	0.825			
postIOP (mmHg)			14.8 ± 4.7	15.5 ± 5.0	14.8 ± 4.8	14.3 ± 4.2	0.114			
Age (years)			56.7 ± 14.4	63.6 ± 14.0	55.5 ± 13.8	51.0 ± 12.5	< 0.001 ***	< 0.001 ***	< 0.001 ***	0.002 **
MD (dB)			-8.9 ± 7.8	-9.7 ± 8.3	-8.2 ± 7.7	-8.9 ± 7.3	0.208			
PSD (dB)			8.0 ± 4.4	7.8 ± 4.5	7.8 ± 4.6	8.4 ± 4.1	0.469			
Total cpRNFLT (μm)			71.4 ± 17.5	69.8 ± 17.3	73.4 ± 17.6	71.2 ± 17.5	0.257			
Quadrant cpRNFLT (μm)	4_Temporal		64.5 ± 20.5	58.3 ± 14.5	67.3 ± 22.4	67.8 ± 22.5	< 0.001 ***	0.001 **	< 0.001 ***	0.927
	4_Superior		85.6 ± 27.3	86.4 ± 26.4	87.6 ± 27.1	82.9 ± 28.3	0.312			
	4_Nasal		58.0 ± 16.5	57.4 ± 14.6	57.6 ± 15.0	59.0 ± 19.5	0.866			
	4_Inferior		77.6 ± 28.5	77.2 ± 29.8	80.9 ± 27.9	74.8 ± 27.6	0.159			
12-sector clockwisecpRNFLT (μm)	Temporal	8 o’clock	60.9 ± 24.7	55.0 ± 16.3	65.3 ± 29.7	62.4 ± 25.1	0.003 **	0.003 **	0.032 *	0.843
		9 o’clock	61.1 ± 19.2	55.3 ± 12.3	62.7 ± 18.9	65.4 ± 23.2	< 0.001 ***	< 0.001 ***	< 0.001 ***	0.668
		10 o’clock	71.5 ± 28.6	64.7 ± 21.9	74.0 ± 29.7	75.8 ± 32.07	0.006 **	0.029 *	0.009 **	0.943
	Superior	11 o’clock	87.9 ± 39.4	86.2 ± 35.5	93.8 ± 41.3	83.87 ± 40.7	0.107			
		12 o’clock	85.8 ± 31.5	89.5 ± 32.2	86.1 ± 29.5	81.8 ± 32.4	0.120			
		1 o’clock	83.1 ± 27.3	83.5 ± 25.2	82.8 ± 25.9	83.1 ± 30.5	0.976			
	Nasal	2 o’clock	65.4 ± 22.0	67.2 ± 20.2	64.7 ± 21.6	64.5 ± 24.1	0.637			
		3 o’clock	53.0 ± 17.5	50.7 ± 14.6	52.2 ± 13.7	55.9 ± 22.4	0.077			
		4 o’clock	55.6 ± 17.5	54.4 ± 15.0	55.9 ± 16.3	56.6 ± 20.7	0.586			
	Inferior	5 o’clock	76.4 ± 24.6	77.0 ± 24.9	76.8 ± 23.7	75.4 ± 25.2	0.920			
		6 o’clock	83.3 ± 35.8	83.1 ± 37.4	88.1 ± 35.5	78.8 ± 33.9	0.079			
		7 o’clock	73.1 ± 39.7	71.5 ± 39.5	77.8 ± 40.9	70.1 ± 38.5	0.086			
Disc topography	Disc area		2.06 ± 0.75	1.96 ± 0.48	2.00 ± 0.61	2.21 ± 1.03	0.286			
	Cup area		1.52 ± 0.78	1.51 ± 0.59	1.41 ± 0.62	1.65 ± 1.04	0.174			
	Rim area		0.54 ± 0.42	0.45 ± 0.33	0.60 ± 0.48	0.56 ± 0.43	0.006 **	0.006 **	0.062	0.709
	Cup volume		0.43 ± 0.36	0.45 ± 0.30	0.42 ± 0.34	0.44 ± 0.44	0.112			
	Rim volume		0.05 ± 0.07	0.04 ± 0.05	0.06 ± 0.08	0.06 ± 0.06	< 0.001 ***	0.002 **	0.008 **	0.865
	C/D area ratio		0.72 ± 0.20	0.76 ± 0.19	0.69 ± 0.20	0.71 ± 0.21	0.008 **	0.007 **	0.088	0.681
	Linear C/D ratio		0.84 ± 0.14	0.86 ± 0.14	0.82 ± 0.13	0.83 ± 0.15	0.008 **	0.006 **	0.093	0.661
	Vertical C/D ratio		0.86 ± 0.15	0.88 ± 0.14	0.85 ± 0.14	0.86 ± 0.16	0.022 *	0.019 *	0.742	0.132
	Disc diameter (V)		1.66 ± 0.25	1.62 ± 0.18	1.64 ± 0.21	1.71 ± 0.34	0.025 *	0.992	0.036 *	0.071
	Disc diameter (H)		1.57 ± 0.31	1.53 ± 0.21	1.55 ± 0.27	1.61 ± 0.40	0.351			
Macular GCC thickness (μm)	Total		78.0 ± 14.7	80.3 ± 13.8	82.3 ± 14.3	77.3 ± 15.6	0.049 *	0.604	0.303	0.039 *
	Superior		83.5 ± 17.0	84.5 ± 15.7	85.5 ± 16.8	80.6 ± 18.2	0.119			
	Inferior		76.5 ± 15.6	76.2 ± 15.8	79.1 ± 15.1	74.1 ± 15.6	0.017 *	0.211	0.471	0.013 *

Asterisks: “*” indicates *p* < 0.05, “**” *P* < 0.01, and “***” *p* < 0.001. *Mid* Middle, *cIOP* Corrected pre-treatment intraocular pressure, *CCT* Central corneal thickness, *BAP* Biological antioxidant potential, *AL* Axial length, *dBP* Dilated blood pressure, *cMT* Corrected mean blur rate in the tissue area of the optic nerve, *postIOP* Post-treatment IOP with applanation tonography, *MD* Mean deviation, measured with the Humphrey visual field analyzer, *PSD* Pattern standard deviation, *C/D* Cup-disc ratio

The dBP subgroups had significant differences in sex, cIOP, BAP, postIOP and MD (Table 4). PreIOP and postIOP in the high dBP group were significantly higher than in the low dBP group and BAP and MD were significantly lower than in the low dBP group. The three groups had significantly different total, superior and inferior cpRNFLT and cpRNFLT in the 6, 7, 10, 11, and 12 o’clock sectors. CpRNFLT in the superior and inferior quadrants and cpRNFLT in the 6, 10, 11 and 12 o’clock sectors were significantly lower in the high dBP group than in the low dBP group. There were no differences in disc area, cup volume, or disc diameter, but in the high dBP group, rim area and rim volume were smaller and the vertical C/D ratio was larger than in the low dBP group. Total, superior, and inferior macular GCC thickness was significantly lower in the high dBP group than in the low dBP group.

**Table 4:** Characteristics of glaucoma patients divided into three groups based on low, medium, or high dBP

Variable			Total (reference) (*n* = 458)	Low dBP (*n* = 153)	Mid dBP (*n* = 152)	High dBP (*n* = 153)	*P* value	*p* value of pairwise comparison test		
								Low—Mid	Low—High	Mid—High
dBP (mmHg)			76.8 ± 13.6	62.9 ± 6.4	75.9 ± 2.9	91.4 ± 10.0				
Male / female			244 / 214	67 / 86	85 / 67	92 / 61	0.012 *			
cIOP (mmHg)			18.8 ± 7.0	17.4 ± 5.3	18.7 ± 6.4	20.4 ± 8.4	< 0.001 ***	0.075	< 0.001 ***	0.193
CCT (μm)			516.6 ± 35.7	518.1 ± 33.9	518.0 ± 38.9	513.7 ± 34.2	0.418			
BAP (µmol/L)			2127.3 ± 284.9	2170.9 ± 251.3	2153.1 ± 195.1	2058.7 ± 368.2	0.027 *	0.507	0.024 *	0.225
AL (mm)			25.4 ± 2.1	25.2 ± 1.6	25.5 ± 1.8	25.7 ± 2.0	0.082			
cMT (arbitrary units)			9.9 ± 2.6	10.0 ± 2.7	10.2 ± 2.6	9.7 ± 2.6	0.327			
postIOP (mmHg)			14.8 ± 4.7	13.9 ± 3.7	14.8 ± 4.7	15.8 ± 5.3	< 0.001 ***	0.227	< 0.001 ***	0.103
Age (years)			56.7 ± 14.4	54.3 ± 17.0	56.8 ± 13.5	58.9 ± 11.9	0.046 *	0.485	0.043 *	0.313
MD (dB)			-8.9 ± 7.8	-7.5 ± 7.0	-8.8 ± 7.6	-10.5 ± 8.3	0.006 **	0.358	0.004 **	0.166
PSD (dB)			8.0 ± 4.4	7.4 ± 4.2	8.0 ± 4.6	8.6 ± 4.3	0.051			
Total cpRNFLT (μm)			71.4 ± 17.5	73.7 ± 16.8	72.1 ± 18.1	68.6 ± 17.3	0.049 *	0.449	0.038 *	0.427
Quadrant cpRNFLT (μm)	4_Temporal		64.5 ± 20.5	66.7 ± 21.0	65.1 ± 19.3	61.7 ± 21.1	0.060			
	4_Superior		85.6 ± 27.3	90.0 ± 26.4	85.1 ± 27.6	81.8 ± 27.5	0.026 *	0.229	0.022 *	0.516
	4_Nasal		58.0 ± 16.5	57.7 ± 15.5	58.2 ± 16.7	58.0 ± 17.4	1.000			
	4_Inferior		77.6 ± 28.5	80.3 ± 28.4	80.1 ± 30.7	72.5 ± 25.7	0.033 *	0.842	0.034 *	0.134
12-sector clockwisecpRNFLT (μm)	Temporal	8 o’clock	60.9 ± 24.7	62.3 ± 24.4	62.0 ± 21.2	58.4 ± 27.8	0.124			
		9 o’clock	61.1 ± 19.2	62.2 ± 18.8	61.1 ± 20.4	60.1 ± 18.3	0.388			
		10 o’clock	71.5 ± 28.6	75.5 ± 29.1	72.5 ± 28.5	66.7 ± 27.6	0.017 *	0.676	0.019 *	0.097
	Superior	11 o’clock	87.9 ± 39.4	94.5 ± 40.1	86.9 ± 40.1	82.3 ± 37.1	0.036 *	0.257	0.029 *	0.598
		12 o’clock	85.8 ± 31.5	90.0 ± 31.3	85.7 ± 30.3	81.6 ± 32.4	0.021 *	0.435	0.019 *	0.212
		1 o’clock	83.1 ± 27.3	85.5 ± 27.4	82.7 ± 26.4	81.3 ± 27.9	0.409			
	Nasal	2 o’clock	65.4 ± 22.0	65.5 ± 21.3	64.8 ± 21.9	66.0 ± 22.9	0.903			
		3 o’clock	53.0 ± 17.5	51.9 ± 14.4	54.2 ± 20.2	52.8 ± 17.3	0.784			
		4 o’clock	55.6 ± 17.5	55.8 ± 16.2	55.7 ± 18.1	55.4 ± 18.3	0.922			
	Inferior	5 o’clock	76.4 ± 24.6	78.2 ± 24.7	77.0 ± 25.4	74.0 ± 23.6	0.252			
		6 o’clock	83.3 ± 35.8	86.1 ± 34.4	86.2 ± 38.0	77.6 ± 34.4	0.048 *	0.899	0.043 *	0.177
		7 o’clock	73.1 ± 39.7	76.6 ± 42.3	77.1 ± 40.7	65.8 ± 34.9	0.027 *	0.987	0.064	0.044 *
Disc topography	Disc area		2.06 ± 0.75	2.10 ± 0.67	2.02 ± 0.69	2.09 ± 0.88	0.894			
	Cup area		1.52 ± 0.78	1.50 ± 0.73	1.48 ± 0.81	1.58 ± 0.81	0.461			
	Rim area		0.54 ± 0.42	0.55 ± 0.34	0.54 ± 0.37	0.51 ± 0.52	0.032 *	0.819	0.029 *	0.154
	Cup volume		0.43 ± 0.36	0.42 ± 0.32	0.42 ± 0.40	0.46 ± 0.36	0.476			
	Rim volume		0.05 ± 0.07	0.05 ± 0.05	0.05 ± 0.06	0.05 ± 0.09	0.046 *	0.878	0.041 *	0.176
	C/D area ratio		0.72 ± 0.20	0.71 ± 0.19	0.71 ± 0.21	0.74 ± 0.21	0.076			
	Linear C/D ratio		0.84 ± 0.14	0.83 ± 0.13	0.83 ± 0.15	0.85 ± 0.14	0.074			
	Vertical C/D ratio		0.86 ± 0.15	0.85 ± 0.13	0.85 ± 0.16	0.88 ± 0.14	0.037 *	0.757	0.030 *	0.197
	Disc diameter (V)		1.66 ± 0.25	1.66 ± 0.24	1.65 ± 0.24	1.67 ± 0.29	0.861			
	Disc diameter (H)		1.57 ± 0.31	1.58 ± 0.30	1.55 ± 0.28	1.57 ± 0.34	0.750			
Macular GCC thickness (μm)	Total		78.0 ± 14.7	82.2 ± 14.7	81.4 ± 14.4	76.3 ± 14.5	0.004 **	0.585	0.003 **	0.060
	Superior		83.5 ± 17.0	86.4 ± 16.8	84.5 ± 16.6	79.6 ± 17.0	0.002 **	0.400	0.001 **	0.077
	Inferior		76.5 ± 15.6	78.1 ± 16.0	78.3 ± 15.3	73.1 ± 15.12	0.016 *	0.997	0.031 *	0.037 *

Asterisks: “*” indicates *p* < 0.05, “**” *P* < 0.01, and “***” *p* < 0.001. *Mid* Middle, *cIOP* Corrected pre-treatment intraocular pressure, *CCT* Central corneal thickness, *BAP* Biological antioxidant potential, *AL* Axial length, *dBP* Dilated blood pressure, *cMT* Corrected mean blur rate in the tissue area of the optic nerve, *postIOP* Post-treatment IOP with applanation tonography, *MD* Mean deviation, measured with the Humphrey visual field analyzer, *PSD* Pattern standard deviation, *C/D* Cup-disc ratio

The BAP subgroups had significantly different sex and dBP (Table 5). We found that dBP in the low BAP group was significantly higher than the middle and high BAP groups. CpRNFLT in the 10 o’clock sector was significantly different in the three groups. There were no differences in disc topography or macular GCC thickness.

**Table 5:** Characteristics of glaucoma patients divided into three groups based on low, medium, or high BAP

Variable			Total (reference) (*n* = 458)	Low BAP (*n* = 153)	Mid BAP (*n* = 152)	High BAP (*n* = 153)	*P* value			
								Low—Mid	Low—High	Mid—High
BAP (µmol/L)			2127.3 ± 284.9	1849.3 ± 304.2	2159.2 ± 46.8	2372.2 ± 101.3				
Male / female			244 / 214	97 / 56	71 / 81	76 / 77	0.008 **			
cIOP (mmHg)			18.8 ± 7.0	19.0 ± 7.3	19.0 ± 7.4	18.5 ± 6.2	0.894			
CCT (μm)			516.6 ± 35.7	514.5 ± 34.3	515.4 ± 37.9	519.8 ± 34.8	0.114			
AL (mm)			25.4 ± 2.1	25.7 ± 1.9	25.3 ± 1.7	25.4 ± 1.8	0.281			
dBP (mmHg)			76.8 ± 13.6	80.0 ± 15.2	76.0 ± 12.3	74.3 ± 12.8	0.003 **	0.049 *	0.003 **	0.521
cMT (arbitrary units)			9.9 ± 2.6	9.6 ± 2.5	10.2 ± 2.6	10.0 ± 2.7	0.226			
postIOP (mmHg)			14.8 ± 4.7	14.8 ± 5.0	14.9 ± 4.6	14.8 ± 4.5	0.811			
Age (years)			56.7 ± 14.4	58.3 ± 12.8	57.1 ± 13.1	54.8 ± 16.8	0.371			
MD (dB)			-8.9 ± 7.8	-9.4 ± 7.7	-8.0 ± 7.3	-9.4 ± 8.2	0.186			
PSD (dB)			8.0 ± 4.4	8.3 ± 4.4	7.6 ± 4.6	8.2 ± 4.3	0.490			
Total cpRNFLT (μm)			71.4 ± 17.5	70.2 ± 16.8	72.8 ± 17.8	71.3 ± 17.9	0.328			
Quadrant cpRNFLT (μm)	4_Temporal		64.5 ± 20.5	62.1 ± 20.1	66.3 ± 20.8	65.1 ± 20.7	0.149			
	4_Superior		85.6 ± 27.3	82.6 ± 26.3	87.1 ± 27.6	87.2 ± 27.9	0.165			
	4_Nasal		58.0 ± 16.5	58.5 ± 16.4	58.3 ± 15.9	57.2 ± 17.3	0.578			
	4_Inferior		77.6 ± 28.5	77.2 ± 27.4	79.7 ± 28.6	75.9 ± 29.5	0.379			
12-sector clockwisecpRNFLT (μm)	Temporal	8 o’clock	60.9 ± 24.7	61.0 ± 27.4	62.5 ± 24.0	59.1 ± 22.4	0.584			
		9 o’clock	61.1 ± 19.2	58.8 ± 16.7	62.6 ± 18.6	62.0 ± 21.8	0.291			
		10 o’clock	71.5 ± 28.6	66.6 ± 27.0	73.8 ± 27.6	74.2 ± 30.6	0.042 *	0.062	0.090	0.997
	Superior	11 o’clock	87.9 ± 39.4	82.2 ± 36.3	90.1 ± 40.4	91.4 ± 40.9	0.097			
		12 o’clock	85.8 ± 31.5	82.6 ± 30.8	87.6 ± 30.8	87.3 ± 32.8	0.222			
		1 o’clock	83.1 ± 27.3	83.0 ± 26.4	83.5 ± 27.6	82.8 ± 27.9	0.937			
	Nasal	2 o’clock	65.4 ± 22.0	65.7 ± 22.1	65.3 ± 22.9	65.3 ± 21.2	0.972			
		3 o’clock	53.0 ± 17.5	53.3 ± 16.1	52.8 ± 14.6	52.7 ± 21.1	0.703			
		4 o’clock	55.6 ± 17.5	56.6 ± 17.7	56.7 ± 17.2	53.6 ± 17.7	0.181			
	Inferior	5 o’clock	76.4 ± 24.6	77.4 ± 25.5	77.6 ± 25.5	74.2 ± 22.6	0.625			
		6 o’clock	83.3 ± 35.8	82.1 ± 33.3	86.9 ± 36.7	80.9 ± 37.1	0.206			
		7 o’clock	73.1 ± 39.7	72.1 ± 38.0	74.6 ± 38.5	72.6 ± 42.5	0.605			
Disc topography	Disc area		2.06 ± 0.75	2.06 ± 0.69	2.05 ± 0.77	2.06 ± 0.80	0.895			
	Cup area		1.52 ± 0.78	1.53 ± 0.71	1.52 ± 0.78	1.52 ± 0.86	0.643			
	Rim area		0.54 ± 0.42	0.53 ± 0.48	0.53 ± 0.40	0.54 ± 0.38	0.622			
	Cup volume		0.43 ± 0.36	0.45 ± 0.35	0.43 ± 0.32	0.43 ± 0.41	0.747			
	Rim volume		0.05 ± 0.07	0.05 ± 0.08	0.05 ± 0.07	0.05 ± 0.06	0.592			
	C/D area ratio		0.72 ± 0.20	0.73 ± 0.20	0.71 ± 0.22	0.71 ± 0.19	0.535			
	Linear C/D ratio		0.84 ± 0.14	0.84 ± 0.13	0.83 ± 0.16	0.84 ± 0.12	0.513			
	Vertical C/D ratio		0.86 ± 0.15	0.88 ± 0.13	0.85 ± 0.17	0.86 ± 0.13	0.290			
	Disc diameter (V)		1.66 ± 0.25	1.67 ± 0.24	1.66 ± 0.25	1.65 ± 0.27	0.422			
	Disc diameter (H)		1.57 ± 0.31	1.57 ± 0.31	1.56 ± 0.31	1.57 ± 0.31	0.957			
Macular GCC thickness (μm)	Total		78.0 ± 14.7	78.8 ± 14.4	80.5 ± 15.2	80.7 ± 14.6	0.557			
	Superior		83.5 ± 17.0	81.4 ± 16.2	84.5 ± 18.1	84.6 ± 16.6	0.195			
	Inferior		76.5 ± 15.6	76.3 ± 15.8	76.4 ± 15.4	76.7 ± 15.8	0.856			

Asterisks: “*” indicates *p* < 0.05, “**” *P* < 0.01. *Mid* Middle, *cIOP* Corrected pre-treatment intraocular pressure, *CCT* Central corneal thickness, *BAP* Biological antioxidant potential, *AL* Axial length, *dBP* Dilated blood pressure, *cMT* Corrected mean blur rate in the tissue area of the optic nerve, *postIOP* Post-treatment IOP with applanation tonography, *MD* Mean deviation, measured with the Humphrey visual field analyzer, *PSD* Pattern standard deviation, *C/D* Cup-disc ratio

The cMT subgroups had significant differences in sex and postIOP (Table 6). PostIOP in the high cMT group was significantly higher than in the low and middle cMT groups. The three groups had significantly different total, temporal, superior, and inferior cpRNFLT and cpRNFLT in the 5, 8, 9 and 10 o’clock sectors. CpRNFLT in the 8, 9, and 10 o’clock sectors was significantly higher in the high cMT group. In the high cMT group, cup area, cup volume, linear C/D ratio, and vertical C/D ratio were smaller than in the low and middle cMT groups. Total, superior, and inferior macular GCC thickness was significantly higher in the high cMT group.

**Table 6:** Characteristics of glaucoma patients divided into three groups based on low, medium, or high cMT

Variable			Total (reference) (*n* = 458)	Low MT (*n* = 153)	Mid MT (*n* = 152)	High MT (*n* = 153)	*P* value			
								Low—Mid	Low—High	Mid—High
cMT (arbitrary units)			9.9 ± 2.6	7.4 ± 1.6	9.8 ± 1.3	12.6 ± 1.8				
Male / female			244 / 214	95 / 58	83 / 69	66 / 87	0.004 **			
cIOP (mmHg)			18.8 ± 7.0	18.5 ± 6.6	18.9 ± 7.4	19.1 ± 7.0	0.730			
CCT (μm)			516.6 ± 35.7	517.1 ± 35.1	512.4 ± 38.5	520.2 ± 33.1	0.160			
BAP (µmol/L)			2127.3 ± 284.9	2140.1 ± 251.4	2127.0 ± 299.1	2114.9 ± 303.6	0.966			
AL (mm)			25.4 ± 2.1	25.7 ± 1.7	25.5 ± 1.9	25.2 ± 1.9	0.087			
dBP (mmHg)			76.8 ± 13.6	75.8 ± 13.7	77.6 ± 14.2	76.9 ± 13.1	0.755			
postIOP (mmHg)			14.8 ± 4.7	14.5 ± 4.7	14.4 ± 4.5	15.6 ± 4.7	0.007 **	0.994	0.018 *	0.016 *
Age (years)			56.7 ± 14.4	55.1 ± 15.9	57.1 ± 13.9	57.9 ± 13.2	0.370			
MD (dB)			-8.9 ± 7.8	-9.6 ± 8.7	-9.1 ± 7.4	-8.1 ± 7.1	0.443			
PSD (dB)			8.0 ± 4.4	7.6 ± 4.2	8.5 ± 4.4	9.0 ± 4.6	0.205			
Total cpRNFLT (μm)			71.4 ± 17.5	69.4 ± 19.5	70.1 ± 16.0	74.8 ± 16.4	0.004 **	0.970	0.016 *	0.007 **
Quadrant cpRNFLT (μm)	4_Temporal		64.5 ± 20.5	62.6 ± 21.5	61.8 ± 19.5	69.1 ± 19.9	< 0.001 ***	0.996	0.003 **	< 0.001 ***
	4_Superior		85.6 ± 27.3	82.2 ± 29.1	84.2 ± 25.7	90.4 ± 26.4	0.013 *	0.809	0.018 *	0.062
	4_Nasal		58.0 ± 16.5	57.2 ± 19.0	59.1 ± 15.3	57.8 ± 15.2	0.692			
	4_Inferior		77.6 ± 28.5	75.7 ± 30.1	75.1 ± 26.3	81.9 ± 28.6	0.043 *	0.997	0.098	0.059
12-sector clockwisecpRNFLT (μm)	Temporal	8 o’clock	60.9 ± 24.7	60.1 ± 28.9	57.8 ± 21.6	64.7 ± 22.5	0.002 **	0.835	0.022 *	0.003 **
		9 o’clock	61.1 ± 19.2	59.3 ± 20.3	59.0 ± 17.7	65.0 ± 18.9	< 0.001 ***	0.998	0.002 **	0.003 **
		10 o’clock	71.5 ± 28.6	68.5 ± 29.8	68.6 ± 26.9	77.4 ± 28.3	0.002 **	0.931	0.005 **	0.010 **
	Superior	11 o’clock	87.9 ± 39.4	84.4 ± 39.5	84.5 ± 38.3	94.7 ± 39.6	0.022 *	0.985	0.040 *	0.051
		12 o’clock	85.8 ± 31.5	82.1 ± 32.7	84.6 ± 29.8	90.7 ± 31.5	0.045 *	0.646	0.046 *	0.205
		1 o’clock	83.1 ± 27.3	80.3 ± 29.8	83.6 ± 25.6	85.6 ± 26.1	0.171			
	Nasal	2 o’clock	65.4 ± 22.0	64.3 ± 24.1	67.0 ± 20.5	65.0 ± 21.3	0.486			
		3 o’clock	53.0 ± 17.5	52.4 ± 21.8	54.2 ± 15.4	52.3 ± 14.4	0.717			
		4 o’clock	55.6 ± 17.5	54.7 ± 18.7	56.2 ± 18.0	56.0 ± 15.8	0.715			
	Inferior	5 o’clock	76.4 ± 24.6	72.4 ± 25.1	76.4 ± 24.6	80.4 ± 23.5	0.005 **	0.226	0.003 **	0.285
		6 o’clock	83.3 ± 35.8	80.5 ± 36.8	81.5 ± 33.7	87.8 ± 36.4	0.115			
		7 o’clock	73.1 ± 39.7	74.1 ± 41.8	67.5 ± 35.1	77.5 ± 41.2	0.091			
Disc topography	Disc area		2.06 ± 0.75	2.29 ± 0.89	2.04 ± 0.60	1.84 ± 0.68	< 0.001 ***	0.015 *	< 0.001 ***	< 0.001 ***
	Cup area		1.52 ± 0.78	1.78 ± 0.96	1.52 ± 0.62	1.26 ± 0.64	< 0.001 ***	0.097	< 0.001 ***	< 0.001 ***
	Rim area		0.54 ± 0.42	0.51 ± 0.46	0.52 ± 0.39	0.57 ± 0.42	0.128			
	Cup volume		0.43 ± 0.36	0.53 ± 0.47	0.43 ± 0.29	0.34 ± 0.26	< 0.001 ***	0.186	< 0.001 ***	0.012 *
	Rim volume		0.05 ± 0.07	0.05 ± 0.09	0.05 ± 0.06	0.06 ± 0.06	0.007 **	0.676	0.008 **	0.057
	C/D area ratio		0.72 ± 0.20	0.75 ± 0.21	0.74 ± 0.18	0.67 ± 0.21	< 0.001 ***	0.450	< 0.001 ***	0.010 **
	Linear C/D ratio		0.84 ± 0.14	0.85 ± 0.14	0.85 ± 0.13	0.81 ± 0.15	< 0.001 ***	0.463	< 0.001 ***	0.010 *
	Vertical C/D ratio		0.86 ± 0.15	0.88 ± 0.14	0.88 ± 0.14	0.83 ± 0.15	< 0.001 ***	0.912	< 0.001 ***	< 0.001 ***
	Disc diameter (V)		1.66 ± 0.25	1.75 ± 0.30	1.65 ± 0.21	1.57 ± 0.22	< 0.001 ***	0.006 **	< 0.001 ***	< 0.001 ***
	Disc diameter (H)		1.57 ± 0.31	1.65 ± 0.34	1.56 ± 0.27	1.48 ± 0.29	< 0.001 ***	0.023 *	< 0.001 ***	0.004 **
Macular GCC thickness (μm)	Total		78.0 ± 14.7	77.2 ± 16.0	78.3 ± 13.5	84.4 ± 13.6	< 0.001 ***	0.452	< 0.001 ***	< 0.001 ***
	Superior		83.5 ± 17.0	79.5 ± 17.3	82.2 ± 16.4	88.7 ± 16.1	< 0.001 ***	0.252	< 0.001 ***	< 0.001 ***
	Inferior		76.5 ± 15.6	75.0 ± 16.6	74.3 ± 14.2	80.0 ± 15.5	0.002 **	1.000	0.009 **	0.006 **

Asterisks: “*” indicates *p* < 0.05, “**” *P* < 0.01, and “***” *p* < 0.001. *Mid* Middle, *cIOP* Corrected pre-treatment intraocular pressure, *CCT* Central corneal thickness, *BAP* Biological antioxidant potential, *AL* Axial length, *dBP* Dilated blood pressure, *cMT* Corrected mean blur rate in the tissue area of the optic nerve, *postIOP* Post-treatment IOP with applanation tonography, *MD* Mean deviation, measured with the Humphrey visual field analyzer, *PSD* Pattern standard deviation, *C/D* Cup-disc ratio

## Discussion

Glaucoma is a multifactorial disease with a variety of reported risk factors. We previously showed that it was possible to adjust OBF measurements with a multi-regression analysis and succeeded in creating an OBF index by adjusting for confounding factors [17]. Here, we expanded this approach to include five other risk factors with different data sets. No previous report has measured these risk factors quantitatively in the same cases. Here, we measured each risk factor in a hospital-based group of 458 glaucoma cases and investigated characteristics of the patients. For each of the risk factors, we performed an independent analysis that divided the patients into three groups based on whether the measured value was low, middle, or high. We then compared structural characteristics and susceptibility area among these groups. Our findings suggest that patients with specific risk factors have unique phenotypes, and that each risk factor is associated with specific types of damage, both in the macula and the optic disc. For example, the patients with high cIOP, low CCT, high AL, and high dBP had a greater loss of macular GCC thickness, which is associated with a lower quality of life. Furthermore, the patients with high cIOP, low CCT, high dBP, and low cMT had a greater total loss of cpRNFLT, and the patients with high cIOP, low AL, and low cMT had a larger C/D area ratio. Therefore, quantifying specific risk factors may help with the classification of specific glaucoma types and clarify the role that different disease mechanisms play in individual glaucoma patients, enabling the future development of IOP-independent treatments for glaucoma.

High IOP is an evidence-based risk factor for glaucoma and the sole modifiable risk factor in glaucoma treatment. In this study, HFA MD was significantly worse in the high cIOP group than the low cIOP group when these groups had similar age and peripapillary cpRNFLT damage in the temporal, superior, and inferior quadrants with large cupping. The direct pathophysiological effect of high IOP remains unclear. High IOP influences the curvature of the lamina cribrosa (LC) [24]. Deformation of the LC induces compression of the RGC axons and of the vessels, both in the optic nerve head and the choroid [25]. In vitro findings show that cultivated astrocytes have a significant gene response to high pressure [26], but that cultivated RGCs do not, suggesting that high IOP induces RGC death via glia-neuronal interaction. Thus, high IOP simultaneously induces axonal damage and lowers OBF by causing deformation of the LC in patients with glaucoma, making high IOP a critical risk factor for glaucoma.

In this study, we found that glaucoma cases with low CCT had lower temporal cpRNFLT and had high CCT with a smaller cup volume. CCT is highly heritable, and low CCT is an independent risk factor for the progression of ocular hypertension to open-angle glaucoma [27]. However, whether low CCT has a negative effect in glaucoma-induced RGC death remains unclear. Previously, cases with low CCT have been found to have low IOP, as measured with Goldmann applanation tonometry [28], which influences the IOP-dependent degeneration pathway. Another hypothesis is that low CCT is related to low-strength collagen in the sclera, cornea, and LC. The strength of the sclera plays a critical role in IOP-induced deformation of the LC [29, 30], and patients with lower corneal deformation amplitude show greater LC depth, greater cup area, a deeper cup, and smaller peripapillary atrophy zone than those with higher corneal deformation amplitude [31]. Thus, a significant body of evidence suggests that properties of the cornea affect the situation of the optic nerve head and influence a patient’s vulnerability to glaucomatous changes.

Myopia has been described as an evidence-based risk factor for glaucoma [6]. Patients with higher myopia have been reported to be more likely to have OAG (OR, 1.59, 95% CI, 1.33–1.91; OR, 2.92, 95% CI, 1.89–4.52 for low, moderate, and high myopia, respectively) [32]. Even though a tilted optic disc has been found to have no statistically significant association with glaucoma progression, greater optic disc torsion is associated with reduced glaucoma deterioration risk, as assessed by functional progression [33]. The prevalence of myopia is higher in East Asia [34], and because NTG is the most common subtype of OAG in Asia [35] there may be an ethnic bias. Elongated AL induces low OBF [22], lamina defects [36], and thinning of the LC [37]. Furthermore, elongated AL induces enlargement of the peripapillary atrophy zone, which results in damage to central visual function [38]. In this study, macular GCC was thinner in glaucoma cases with high AL than in those with middle AL. Interestingly, we found that temporal cpRNFLT was also lower in cases with low AL than in normal subjects. Cases with low AL were significantly older and had higher dBP in the three groups, while patients with POAG and low AL had higher 24-h IOP fluctuation [39] than patients with myopia. These characteristics increase vulnerability to change in temporal cpRNFLT, but the precise reason for this is still unclear. Further research is needed to clarify the mechanism of macular damage in eyes with a short AL.

We found that the high systemic dBP group was more likely to have severe visual field defects, low total, superior, and inferior cpRNFLT, and low macular GCC thickness than the low dBP group. The group of patients with high dBP was significantly older with significantly lower BAP, suggesting that high dBP is related to systemic anti-oxidant levels. We also found that cIOP was high in the high dBP group. Thus, high dBP was related to older age and high IOP, resulting in more severe glaucoma. Previously, both low and high systemic blood pressure have been reported to be related to low OBF and the presence of glaucoma [12], while lower mean systemic blood pressure has been shown to be a risk factor for a smaller rim area in the superior optic disc  [40]. Population-based epidemiological studies have found a strong relationship between low ocular perfusion pressure and OAG prevalence, incidence, and progression [5]. Subjects with Flammer syndrome, a type of primary vascular dysregulation, tend to have increased risk for NTG and a higher prevalence of optic disc hemorrhages [41]. Moreover, a study of nocturnal blood pressure changes found that subjects who were non-dippers or extreme dippers were at risk for progressive visual field loss in glaucoma [42]. These findings suggest that abnormalities in systemic BP, both high and low BP, are a risk factor for glaucoma, and point to the importance of measuring systemic BP in daily clinical practice. Furthermore, we need to conduct a study that includes the continuous measurement of systemic BP in order to determine the relationship between fluctuations in systemic BP and glaucoma progression.

The critical contribution of oxidative stress to glaucomatous damage has been previously described [1]. The total antioxidant status of the blood has been shown to be lower in OAG patients than control subjects [43]. Furthermore, younger male glaucoma cases with low antioxidant levels tend to have worse visual field defects [23]. In this study, there were more male glaucoma patients in the low BAP group. Oxidative stress has also previously been shown to be related to dysfunctions of the capillaries in the optic nerve head [44] and cases with low BAP have been shown to have higher dBP. Among structural changes, the loss of cpRNFLT was only detected in the 10 o’clock area; the cause of this vulnerability to oxidative stress remains unclear. Antioxidant treatments, such as a diet high in nitrates and green leafy vegetables, is associated with a lower POAG risk, particularly POAG with early paracentral VF loss at diagnosis [45]. A total of 33 interventional trials suggest that flavonoids exert a beneficial effect in glaucoma [46]. Thus, we developed several solutions based on these findings, such as dietary supplementation and boosted vegetable intake. We have also demonstrated that hesperidin has an antioxidant property in both mice [47] and humans [48].

Previously, we examined 533 patients with glaucoma and found that patients with low OBF were characterized by a larger cup-disc ratio and higher susceptibility to damage in the temporal disc and the macular area [17]. In this study, we found that cases with high OBF had higher total cpRNFLT, temporal and superior cpRNFLT, and macular GCC thickness. Previously, NTG eyes that were undergoing medical treatment and had greater 24-h mean ocular perfusion pressure fluctuations have been shown to have faster central visual field defect progression [49]. The blood supply of the optic nerve originates in a branch of the post ciliary artery and the circle of Zinn-Haller [50, 51]. Therefore, disturbances in these vessels may play a causative role in glaucomatous optic neuropathy. LSFG enables us to measure the deep layers of the optic nerve head around the LC [20]. Recently, we showed that reduced LSFG-measured tissue blood flow in the ONH was already detectible in PPG [14, 15] and that low OBF could predict the progression of glaucoma [16]. We have also clearly shown, with retrospective and prospective data, that low OBF is a predictor of temporal and superior cpRNFLT loss, but not inferior cpRNFLT loss [18, 52]. Thus, decreased OBF in the ONH can be either the cause or the result of glaucoma.

This study had several limitations. First, it was cross-sectional and included subjects from only a single hospital and a single ethnicity (Japanese). Second, when we investigated structural differences and their relationships with various risk factors in glaucoma patients, we found that it might have been better to exclude patients with myopia due to the variety of appearances a myopic disc can have. However, in Asia, mild to moderate myopia is a major risk factor for glaucoma [2], making it difficult to completely exclude myopia from this study. Therefore, we only excluded cases with severe myopia, to minimize the effect of myopia. Third, in Japan, older generations tend to have emmetropia and younger generations tend to have myopia. This generational bias might also have influenced our results. In order to minimize treatment bias, we recruited as many subjects as possible (over 450 eyes). In the near future, we would like to perform a study that classifies cases with constant parameters, such as genetics, that would result in a more stable classification of glaucoma. Fourth, this study was part of ongoing exploratory research with the goal of classifying glaucoma based on specific risk factors and developing new glaucoma treatment algorithms. In this study, as a first step, we investigated the relationship between measurable risk factors and damage type in glaucoma, including in the macula and optic nerve head. Our findings revealed many differences and associations, but did not allow us to form a clear hypothesis on expected risk profiles. Further study is needed to obtain succinct, conclusive findings that will benefit health care providers and patients.

## Conclusion

In conclusion, this study identified subgroups of glaucoma patients with differing risk factors. These patients tended to have unique phenotypes, and we found that they have risk factors that were IOP-independent. Our findings suggest that there is at least one category of glaucoma patients for whom different risk factors contribute to the pathophysiology of glaucoma, and that these patients may benefit from treatments that target these risk factors. We consider that mechanism-dependent treatment is a reasonable approach to glaucoma care, and that our approach to the clinical measurement of risk factors may open new avenues for the individual treatment of glaucoma.

## Data Availability

The datasets used and/or analyzed during the current study are available from the corresponding author on reasonable request.
